# Assessment of the quality of root canal fillings performed on extracted teeth by undergraduate dental students in a sample from Saudi Arabia

**DOI:** 10.1186/s12909-024-05136-4

**Published:** 2024-02-19

**Authors:** Ahmed A. Madfa, Moazzy I. Almansour, Saad M. Al-Zubaidi, Ahmed H. Albaqawi, Saleh A. Almeshari, Anas A. Khawshhal, Rehab H. Alshammari

**Affiliations:** Department of Restorative Dental Science, College of Dentistry, University of Ha’il, Kingdom of Saudi Arabia, Ha’il, Kingdom of Saudi Arabia

**Keywords:** Dental students, Education, Endodontics, Root canal obturation, Saudi Arabia

## Abstract

**Background:**

The educational process in the field of endodontics commences with preclinical exercises to enhance students’ proficiency in cleaning, shaping, and performing root canal fillings. Therefore, this study aimed to radiographically evaluate the technical quality of root canal fillings performed by preclinical students on extracted teeth at the College of Dentistry, University of Ha’il, Saudi Arabia.

**Methods:**

A total of 788 extracted human teeth received root canal treatment by undergraduate students. The samples were then gathered and radiographically assessed using the three quality criteria of length, density, and taper. The category of root canal fillings was classified as either acceptable or unacceptable. The criteria for evaluating the acceptability of filling quality were defined based on the presence of adequate length, density, and taper. The effectiveness of root canal fillings was also evaluated in relation to the tooth type, sex, and treatment year. The agreement between the examiners was evaluated using Cohen’s kappa test, and the relationship between the research variables was determined using the chi-squared test. The significance threshold was set at 0. 05.

**Results:**

The overall quality of root filling was determined to be satisfactory in 532 (67.5%) of 788 endodontically treated extracted teeth. The majority of the research sample (88.1%) had enough length, 89.6% had adequate density, and 86.4% had acceptable taper. The quality of anterior teeth was substantially better than that of posterior teeth (*p* < 0.001). Our findings showed that the quality of root canals was better in 2022 than it had been in earlier years (*p* = 0.001). The three RCT quality criteria differed significantly when compared between sexes (*p* = 0.002).

**Conclusions:**

The quality of the root canal fillings completed by undergraduate students was rated as acceptable. The findings of the research suggest that the implementation of routine assessments to evaluate the technical competence of undergraduate dental students performing root canal treatments could provide significant insights into the efficacy of the curriculum requirements.

## Background

Endodontic treatment is necessary for a sizable part of dental emergencies treated globally, and there is a growing demand to train and educate dental students in this skill so they would be more ready to carry out such treatment with satisfactory outcomes [1, 2]. However, numerous investigations have shown that general dentists do not deliver endodontic care of a high standard [3–4]. Although there may be several contributing causes to this deficiency, a significant portion is ascribed to the inadequate endodontic training given to dental graduates of the majority of colleges [5]. This may be attributed to the continued utilisation of traditional instructional approaches in the majority of dental schools [5]. The European Society of Endodontology (ESE) established recommendations for the undergraduate curriculum in endodontology in 1992 and 2001 to address deficiencies in the curricula of various institutions in Europe and to assure a minimum level of endodontic competency before graduation [6]. In response to several reports that revealed a reduced use of conventional endodontic treatment in European populations [7, 8], a new recommendation was issued in 2013.

The assessment of the technical quality of the obturation process entails the radiographic evaluation of three criteria, as stated by the American Association of Endodontists [12]. These parameters include the measurement of the root canal filling’s length, taper, and density. As per the guidelines established by the ESE on radiographic evaluation of root canal fills, it is imperative to guarantee complete and perfect filling of the root canal, with no presence of gaps or voids between the filling material and the canal wall. Furthermore, it is recommended that the filling be placed at a distance of 0.5-2 mm from the radiographic apex [13].

The endodontic preclinical and clinical phases of dental students’ education have always been crucial to the art and science of dentistry. Students who successfully complete a preclinical program will have the information and abilities needed to transition into clinical settings and to meet the different diagnostic and therapeutic difficulties that are presented in clinical courses [5, 11, 14–16]. Research has already assessed the quality of RCTs completed by students and the significance of preclinical proficiency [17–19]. The investigators discovered that undergraduate students filled root canals with sufficient technical quality at rates ranging from 13 to 79.47%. Dental education can be improved by assessing endodontic student performance [20, 21].

The undergraduate dental students in the College of Dentistry at the University of Ha’il receive instruction in endodontics during the 4th, 5th, and 6th years of their Bachelor of Dental Surgery (BDS) programme. The endodontic programme in our college consists of three primary components: educational lectures and their corresponding learning exercises, preclinical instruction, and clinical instruction. The delivery of lectures and the corresponding learning activities should aim to impart comprehensive knowledge that is essential for the effective implementation of endodontic procedures. The cornerstone and introduction to the specialty is the endodontic preclinical program, which is regarded as a key phase in equipping the student with the core expertise needed to begin providing patients with appropriate endodontic care. Preclinical endodontics (RDS 424), a fourth-year course, is primarily designed to prepare students for identifying, diagnosing, and successfully treating teeth. The foundations of endodontics are highlighted throughout lectures, with an emphasis on the connection between essential clinical and biological concepts. During this course, the students are taught subjects related to endodontic procedures, such as the internal root anatomy, access cavity perpetration, methods of working length determination, and methods of root canal preparation, disinfection, and filling. Dental students receive preclinical instruction throughout the first and second semesters on how to execute RCT on single- and multirooted extracted teeth. For 30 weeks, they complete the technical components of root canal procedures on extracted teeth throughout the course’s 3 practical credit hours and 1 theoretical credit hour of instruction every week. In our dental college, extracted teeth are the most prevalent teaching aids utilized in preclinical training. Training with extracted human teeth has the advantage of clinical condition comparability [5]. The utilization of extracted teeth as a means of instructing preclinical students in the field of endodontics has been widely adopted, providing students with a valuable opportunity to develop their skills prior to engaging in clinical practice with real patients [5, 10]. Students in their fifth year of study who have successfully completed the theory and practical portions of RDS 424 are enrolled in the clinical endodontics (RDS 511) course. It builds on the theoretical portion of the endodontic training from RDS 424, gives students clinical experience, and exposes them to actual patient care. The confluence of clinical and biological concepts is the course’s main focus. Both anterior and posterior teeth with endodontic involvement will be taught to the students. They will also receive training in handling severe injuries and endodontic emergencies. The most complicated cases are handled in the sixth year’s Comprehensive Care Clinics (CCC 600) course, which is in subsequent disciplines.

The examination of the technical proficiency of RCT conducted by undergraduate preclinical dental students has garnered attention in numerous countries characterized by distinct educational systems [5, 10, 22–24]. Regular evaluation of preclinical endodontic student performance results will help to enhance dental education and raise the standard of students’ clinical performance [5, 10]. Few studies involving the assessment of the technical proficiency of preclinical students have been undertaken, despite the significance of completing preclinical RCTs [6, 10, 22, 24, 25]. The importance of enhancing RCTs in Saudi Arabia can be achieved by a focus on the education and training of undergraduate students. However, there have been no prior studies to assess the technical quality of root canal fillings performed by dental students at the University of Ha’il, Saudi Arabia. Therefore, the current study aimed to investigate the technical quality of root canal obturations in extracted teeth conducted by preclinical undergraduate students at the College of Dentistry, University of Hail, Saudi Arabia utilizing radiographic criteria.

## Method

### Study design

This retrospective cross-sectional study was designed to evaluate the technical quality of root canal fillings completed by fourth-year students at the at the College of Dentistry, University of Ha’il during the 2019–2022 academic year. The research encompassed the analysis of a randomly selected sample of 788 endodontically treated teeth’s final radiographs. The teeth used in this study were used for educational purposes during their practice. For the publication of their findings, the students gave their consent. The Research Ethics Committee members decided to exclude the study from ethical review since the teeth were primarily utilized for educational purposes and were essentially recommended for extraction (for orthodontic or periodontal reasons). The work completed by dental students, comprising the preoperative, working length, master cone, and postoperative periapical radiographs, served as the source of the data for this study. The Helsinki Declaration of the World Medical Association guided the conduct of this study.

### Sample size calculation

The sample size was calculated based on Cochran’s formula as follows: N= (Zα^2^ × P (1-P))/D^2^.

Zα, which represents the normal distribution critical value at α/2 (1.96); D, which signifies the degree of precision; P, representing the unacceptable technical quality of root canal fillings (53.3%), as determined by a prior study [22]. The suggested sample size for the study was 384. The final sample was 788 extracted treated teeth.

### Sample selection

A comprehensive assessment was conducted on a total of 788 extracted teeth subsequent to the completion of root canal treatment by dental students. To ensure inclusion, certain criteria needed to be met, including the presence of permanent teeth, radiographs that possess diagnostic utility are characterised by their clarity and absence of distortion, hence enabling accurate interpretation and analysis. This study also includes radiographs that exhibited completely developed roots during the treatment process, as well as the standard set of four radiographs (initial, working length, master cone, and posttreatment). The exclusion criteria encompassed several factors, namely, the absence of any of the standard four radiographs, the presence of radiographs with limited diagnostic efficacy, the existence of calcified root canals, the incomplete formation of roots, the occurrence of internal or external root resorption, and the presence of teeth with highly curved roots. Furthermore, teeth that had intricate anatomical features such as fused roots, merged canals, and C-shaped canals were also excluded from the present study. All extracted teeth were embedded in roots and fixed in an acrylic teaching model.

### Root canal procedure

Carbide round burs were used (SS White Dental, New Jersey, USA) to obtain access to the pulp chamber. The working length was established following the removal of all the remaining pulpal tissues. Most frequently, Gates-Glidden drills (Dentsply Sirona, Tulsa, Oklahoma, USA) nos. 2, 3, and 4 were utilized to enable straight-line access. Radiographs (Digital Dental X-ray Sensor, Dentsply Sirona, USA) were used to calculate working lengths. Stainless steel K-files (Dentsply, Tulsa, Oklahoma, USA) of 0.02 taper were used to prepare all canals using the step-back technique. A syringe was used to irrigate the canals with 1% sodium hypochlorite solution. A cold lateral compaction technique was then used to fill the root canals with 0.02 taper gutta-percha (DiaDent Corp., Chungcheongbuk-do, Korea) and AH-26 resin sealer (Dentsply DeTrey GmbH, Konstanz, Germany). To evaluate the technical quality of the RCTs, postoperative radiographs were analysed.

### Radiographic evaluation

The distribution of teeth treated by students was examined, along with the year of treatment. On an illuminated viewer box in a darkened room, 2.5X magnification was used to view the postoperative radiographs. Before performing the evaluation, the examiner participated in calibration training. Six observers [three endodontists with more than 10 years of experience (AAM, MIA, and SMA) and three with 5 years of experience (SAA, AAK, and RHA)] assessed the quality of the post-obturation radiographs to enhance inter-evaluator reliability. In case of disagreement, the observers engaged in a collective evaluation and discussion, ultimately arriving at a final consensus.

The density of the filling, the taper of the root filling, and the distance from the filling’s end to the radiography apex were used to evaluate the quality of the root fillings [6]. According to the criteria in Table 1, the root canal filling quality was categorized. The scores for each parameter ranged from 1 (acceptable) to 2 (unacceptable). The root canal filling length, density, and taper were evaluated for each tooth. For teeth with several roots, the root with the lowest score was taken into account. The failure of one criterion resulted in the failure of the treatment, and the failure of one root in a tooth with several roots caused the failure of the entire treatment. The agreement between the examiners was evaluated using Cohen’s kappa. The findings showed that all assessment criteria had practically perfect agreement (Kappa value = 0.91) [21].

**Table 1:** Criteria used in this study to evaluate the technical quality of the root canal fillings

Definition	Criteria	Parameters
Length	Acceptable	“Root canal filling 0-2 mm short from the radiographic apex”.
	Unacceptable	“Root canal filling beyond the radiographic apex or root canal filling > 2 mm from the radiographic apex”.
Homogeneity	Acceptable	“Homogeneous root canal filling, good condensation, no visible voids”.
	Unacceptable	“Non-homogeneous root canal filling, poor condensation or voids present”.
Taper	Acceptable	“Consistent and uniform taper from the coronal to apical area with a reflection of the original shape of the canal”.
	Unacceptable	“Non-consistent taper”.

### Statistical analysis

The Statistical Package for the Social Sciences from IBM Co. was used for the data analysis, which included frequency distribution and cross-tabulation. The root canal filling length, density, taper, and overall technical quality were assessed. Chi-square and Fisher’s exact tests were conducted to analyse the association between root canal filling quality and academic year, sex, and tooth type. A *P* value of 0.05 was chosen as the significance level.

## Results

A total of 788 teeth from 436 (55.3%) male students and 352 (44.7%) female students were investigated in this study. The most examined samples were molars, accounting for 35.0% of the total sample, with 276 teeth, followed by anterior and premolar teeth, representing each 32.5% of the sample, with 256 teeth.

**Fig. 1:**
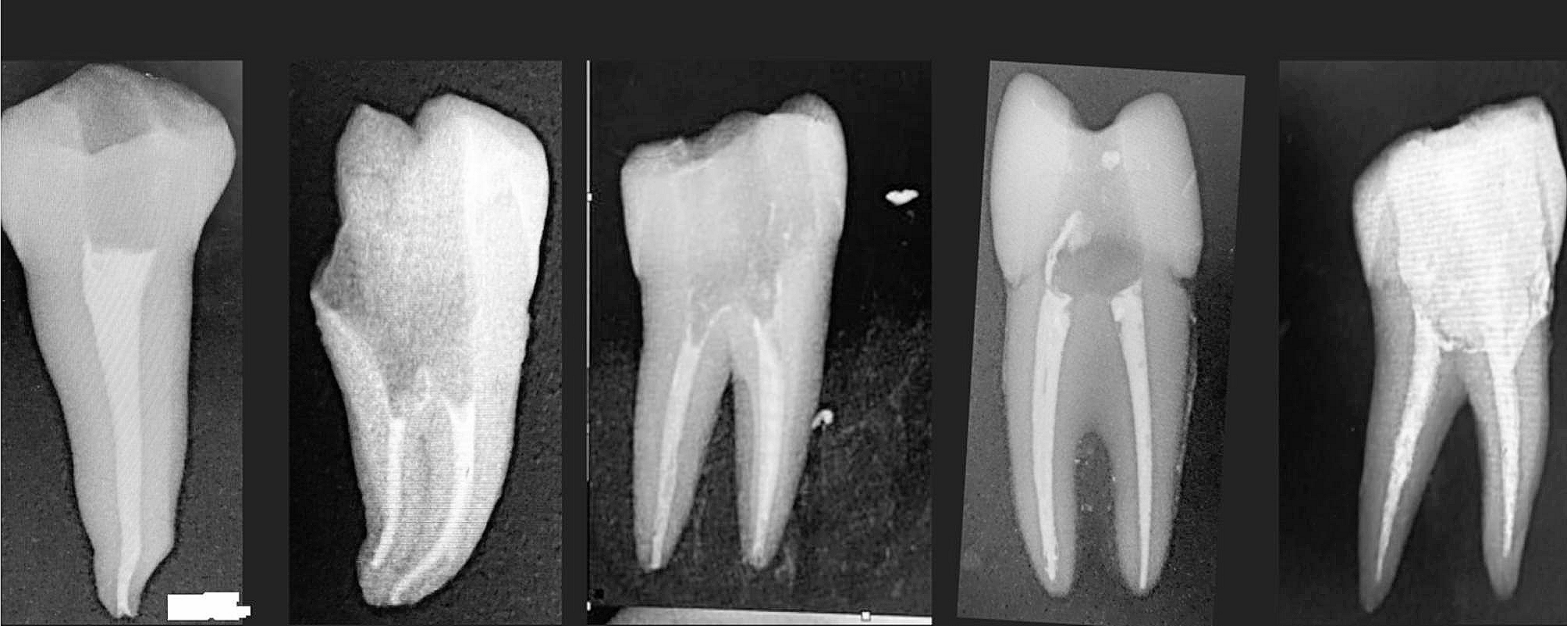
Some radiographic examples of the dental students work

The overall quality of root filling was acceptable in 532 (67.5%) of the research samples, according to the analysis. According to Table 2, the majority of the study sample (88.1%) had enough length, 89.6% had adequate density, and 86.4% had adequate taper. Figure 1 illustrates some radiographic examples of the dental students’ work.

**Table 2:** Quality of root canal fillings based on length, density, and taper criteria

Variables		Frequency	Percent
Length	Acceptable	694	88.1
	Unacceptable	94	11.9
Density	Acceptable	706	89.6
	Unacceptable	82	10.4
Taper	Acceptable	681	86.4
	Unacceptable	107	13.6
Overall filling quality	Acceptable	532	67.5
	Unacceptable	256	32.5

The evaluation of the filling quality of the root canals according to the treatment year, sex, and tooth type is shown in Table 3. The percentage of obturations that were acceptable was highest in 2022 (92.9%), while it was lowest in 2020 (83.8%). The best density quality was reported in 2022 (96.4%), while the worst was noted in 2020 (84.8%). The adequate taper reached its peak in 2022 (91.4%) and its lowest point in 2020 (80.7%). The three RCT quality criteria differed significantly across treatment years (*p* = 0.001).

**Table 3:** Quality of root canal fillings according to treated year, gender, and tooth type

Variable		Length n (%)		Density n (%)		Taper n (%)		Overall filling quality n (%)	
		(1) *	(2) ^+^	(1) *	(2) ^+^	(1) *	(2) ^+^	(1) *	(2) ^+^
Year	2019	167 (84.8)	30 (15.2)	177 (89.8)	20 (10.2)	171 (86.8)	26 (26)	116 (58.9)	81 (41.1)
	2020	165 (83.8)	32 (16.2)	167 (84.8)	30 (15.2)	159 (80.7)	38 (19.3)	112 (56.9)	85 (43.1)
	2021	179 (90.9)	18 (9.1)	172 (87.3)	25 (12.7)	171 (86.8)	26 (13.2)	149 (75.6)	48 (24.4)
	2022	183 (92.9)	14 (7.1)	190 (96.4)	7 (3.6)	180 (91.4)	17 (8.6)	155 (78.7)	42 (21.3)
		*P* = 0.001		*P* = 0.012		*P* = 0.039		*P* = 0.001	
Sexes	Male	366 (83.9)	70 (16.1)	381 (87.4)	55 (12.6)	360 (82.6)	76 (17.4)	275 (63.1)	161 (36.9)
	Female	328 (93.2)	24 (6.8)	325 (92.3)	27 (7.7)	321 (91.2)	31 (8.8)	257 (73.0)	95 (27.0)
		*P* = 0.007		*P* = 0.015		***P = 0 0.001***		*P* = 0.002	
Tooth type	Anterior teeth	242 (94.5)	14 (5.5)	237 (92.6)	19 (7.4)	229 (89.5)	27 (10.5)	187 (73.0)	69 (27.0)
	Premolars	239 (93.4)	17 (6.6)	234 (91.4)	22 (8.6)	235 (91.8)	21 (8.2)	187 (73.0)	69 (27.0)
	Molars	213 (77.2)	63 (22.8)	235 (85.1)	41 (14.9)	217 (78.6)	59 (21.4)	158 (57.2)	118 (42.8)
		*P* < 0.001		***P = 0 0.003***		*P* = 0.001		*P* < 0.001	

(1) * = Acceptable; (2) ^***+***^ ***= Unacceptable***

The There was an adequate length of obturation in 93.2% of teeth treated by female students and in 83.9% of those treated by male students. The quality of density was better in samples treated by females (92.3%) than in those treated by males (87.4%). Additionally, the adequacy of the taper was higher in samples treated by female students (91.2%) than in those treated by males (82.6%). A comparison among sexes showed significant differences in the three criteria of RCT quality (*p* = 0.002).

The adequate length of the obturation was the best in anterior teeth (94.5%), followed by premolars (93.4%) and molars (77.2%). Incisors (92.6%), premolars (91.4%), and molars (85.1%) had the best density quality. Adequate taper was the highest in premolars (91.8%), followed by incisors (89.5%) and molars (78.6%). A comparison among various types of teeth showed significant differences in the three criteria of RCT quality (*p < 0.001*).

## Discussion

The present study investigated the quality of root canal fillings accomplished by preclinical undergraduate dental students at the College of Dentistry, University of Ha’il, Saudi Arabia between 2019 and 2022. The evaluations were conducted utilizing X-ray imaging techniques. Based on current European recommendations and earlier clinical investigations of RCTs conducted by dental students, the radiographic criteria were used to assess the technical qualities of root canal obturation [5, 10, 26]. Radiographic techniques are the basic foundation for the assessment of the quality of RCTs conducted by students in educational institutions [5, 14–16]. The degree of interexaminer variability and intraexaminer repeatability has an impact on how periapical disease is interpreted on radiographs [27, 28]. The degree of interexaminer variability may have an impact on the analysis and interpretation of radiography images [26]. High interexaminer repeatability values in our study (Kappa values = 0.91) confirmed a high degree of evaluation dependability. By encouraging two-dimensional images, this approach implies limits, and the radiographs were not obtained in a completely uniform and repeatable manner.

The satisfactory root canal filling quality as a whole was 67.6%. These results are consistent with the results of previous studies conducted in Saudi Arabia [29, 30] and are comparatively higher than those of other studies conducted in Iran and Saudi Arabia [31, 32]. In another previous study in Saudi Arabia, the technical quality of root canal fillings performed by preclinical dental students was considered acceptable in 46.7% of cases [22]. Some earlier studies revealed success rates of RCT between 3.6 and 54% [9, 10, 31–34]. This observed discrepancies in the outcomes might be ascribed to the differences in educational methodologies. These discrepancies may be also attributable to the different assessment criteria employed in different studies, some of which only utilized two criteria (length and density) [30, 35], the majority of which were carried out by clinical undergraduate students [31–34], and others that only examined one set of teeth [35, 36]. Due to the complexity of the cases, this may have had an impact on the overall quality of the root canal filling. The number of patients and the types of treatments can affect how much practical experience a student gains and how self-efficacious they become in an endodontic clinic [20, 21]. Therefore, it is recommended to begin with RCT cases that are more manageable in terms of difficulty.

The technical difficulty of obturating multirooted teeth is widely recognized, mostly attributed to their anatomical positioning inside the oral cavity and the complex configuration of their root canal system [37]. It has been demonstrated that with time, clinical training improves students’ performance, even though they may still underperform in the obturation of teeth that are placed more posteriorly in the dental arch [38]. As anticipated in the present study, the level of root canal quality observed in the anterior teeth was superior to that observed in the posterior teeth. This finding is consistent with previous research [34]. Our findings are also in line with those of Saatchi et al. [31] and Al-Anesi et al. [10], who found that teeth with fewer roots were of higher quality than teeth with more roots.

Our findings indicated that root canal quality was higher in 2022 than in prior years. This may be because our institution, like other dental colleges, significantly altered the way its curriculum was taught in response to COVID-19 in earlier years. These adjustments were made in accordance with contingency plans established while considering the security of faculty, staff, and students, as well as the continuity and upkeep of the standard of dental education and adherence to the rules and regulations established by governing bodies [39]. The pandemic/epidemic makes it challenging to conduct clinical sessions and provide dental education. However, educators have looked into their options and are modifying their instruction by using online technologies. The students adjusted to the online learning environment and learned what they needed to know through theoretical lectures delivered online and hands-on clinical practice under strict safety precautions.

In our investigation, the results indicated that female students had 73% acceptable obturation, while males had 63.1%. These values in our study were higher than those in another study reported by Agwan et al. [40], which found that females had a 57% adequate obturation length, while in males, that value was 62%. In another previous study in Saudi Arabia, Alosaimi et al. [29] reported that the technical quality of root canal fillings performed by female students was 51.9% compared to 48.1% for male students. This can be elucidated by their extensive expertise in administering root canal procedures.

The challenges of root canal filling, according to Santos et al. [41], include the retention of a suitable apical extension and the adaptation of the master gutta-percha cone in the important apical zone. The greatest significant impact on treatment outcomes was caused by the length or apical extension of the obturation parameter [17]. Comparing the satisfactory cases with those from earlier research by Al-Anesi et al. (53.4%) [10], Balto (67.4%] [34], and Fong et al. (72%) [42], acceptable cases (88.1%) were found in this study for the length parameter. Other authors (86.2%, 89.6%) who conducted an audit at Qassim University in Saudi Arabia observed similar findings [43, 44]. The fact that the students took periapical radiographs to ascertain the suitable working length and to check the master cone’s proper adaptation may be responsible for the study’s high percentage of sufficient length.

Radiographic density [26] is an additional parameter for evaluating potential obturation defects in root canal therapy. Due to microleakage of the root filling, insufficient density may be followed by root canal failure [44]. Improper gutta-percha condensation in the root canal is indicated by inadequate density. A greater incidence of apical periodontitis is brought on by the low density of root fillings [17, 45]. The typical obturation method utilized in this research was the lateral compaction method, which is the method in which dental school students are often instructed [46]. This could have an effect on how frequently voids are found on radiographs. In the current report, an acceptable taper (89.6%) was the metric that was most commonly accomplished among the several parameters used to evaluate the quality of obturation, which is in agreement with Javed et al. (89%) [43] and Fong et al. (90%) [42] but not with Balto et al. (34.9%) [34].

In the current report, an adequate taper was found in 86.4% of the studied sample. Fong et al. [42] found findings that were similar; however, Al-Anesi et al. [10] and Alhablain et al. [44] reported substantially lower frequencies with an acceptable taper (14.2% and 54.1%, respectively). When individual canals were examined for taper adequacy, the placement of the canal had a substantial impact on the results.

The European standards stated that formative and summative evaluation should play a substantial role in competency demonstration [5, 14, 15, 46]. In our institution, the total time allowed for practical sessions was 4 h each week (1 theoretical + 3 practical) or approximately 120 h for the full preclinical endodontics course (30 + 90). This period of time was longer than those recorded in Germany (78 h) and the United Kingdom (60 h) [5, 15]. The ratio of faculty to students plays a significant part in training graduate students at various educational levels to achieve technique-sensitive abilities [5, 14, 15, 46]. In the present study, the student-to-supervisor ratio was 6:1. Sacha et al. [5] found in German-speaking countries that the most favourable faculty-to-student ratio was 1:4, and the worst was 1:38. Mergoni et al. [46] reported in Italy that the staff-to-student ratio during preclinical training ranged from 1:4 to 1:20, with an average ratio of 1:8.9.

The utilization of efficient methods of instruction and appropriate educational resources is a crucial component in facilitating successful teaching and learning endeavours. Numerous factors affect the quality of education, including the amount of time spent on theoretical and practical instruction and training in both preclinical and clinical settings, the supervisor-to-student ratio, the teaching and assessment methodology, instruction materials, etc. [5, 14–16, 46].. Modifications should be made to the preclinical endodontic curriculum to improve the clinical performance of undergraduate students in endodontics. Therefore, it is necessary to modify the endodontics curriculum to incorporate the latest advancements in devices and materials. Certainly, it is imperative to incorporate the instruction of NiTi rotary instrumentation into the dental curriculum for undergraduate students [47]. This particular technique enables a more efficient and precise preparation of root canals, thereby minimizing the occurrence of endodontic mishaps compared to the utilization of stainless-steel hand instruments [48, 49]. Furthermore, the enhancement of preclinical training through the incorporation of new techniques to determine working length has the potential to enhance the clinical proficiency of dental students [50]. Furthermore, it has been shown that employing the vertical compaction technique for root canal filling, as opposed to lateral compaction, and taking measures to prevent the extrusion of the obturation material can result in a more uniform and consistent root canal obturation [51]. The utilization of 3D-printed teeth presents a highly effective method for creating individualized canals and refining psychomotor abilities [16, 46]. The implementation of 3D-printed artificial dental simulators featuring canals with simple and S-shaped configurations is recommended for integration into preclinical training in our college. In addition, the incorporation of virtual reality dental simulators into preclinical training for undergraduate dental students in the field of endodontics represents a great educational resource that has the potential to enhance existing conventional teaching approaches [52]. Furthermore, it is recommended that undergraduate endodontic instruction should include the integration of magnification and ultrasonic devices [14–16].

The radiographic quality of obturation is a significant aspect that influences the results of RCTs; however, it is vital to note that this may not accurately reflect the treatment’s disinfection process. An essential method of quality control in dental schools is an audit of this kind of root canal obturation by undergraduate students. These audits support the idea that the curriculum standards need to be reviewed and assist in uncovering areas of insufficiency.

There are some limitations of this study that should be considered. The radiography database is the only source of information that is available due to the retrospective research design. Additionally, the present study was based on radiographs, which are not a perfect measure of root canal filling quality. Furthermore, because radiographic examination and interpretation have inherent limitations, methodological errors may have been introduced. Radiographs may not have been taken in a manner that is strictly standardized and reproducible to be more precise. Moreover, changes in beam and film angulation have an effect on how the parameters under discussion appear on radiographs. This study was conducted at a single institution, and the results may not be generalizable to other dental schools. In this investigation, we employed rigorous criteria to evaluate the quality of root canal obturation. It is recommended that future studies be undertaken with larger sample sizes and with a broader definition of radiographic acceptable and unacceptable root canal obturations. In future research, it is imperative to investigate the potential impact of students’ theoretical scores of endodontic courses on the overall quality of root canal treatment. It will be very interesting to conduct clinical research with students from the same academic year to track how their endodontic work is progressing and to contrast their preclinical and clinical accomplishments.

## Conclusions

Under the limitations of the present study, it was found that the technical quality of the root canal fillings achieved by undergraduate students was adequate. The technical proficiency of root canal fillings was impacted by COVID-19. The factor that the undergraduate students used best was the length and density of root canal fillings. The quality of the root canal filling was influenced by gender and tooth type. The findings of the present research suggest that the implementation of routine assessments to evaluate the technical competence of undergraduate dental students performing root canal treatments could provide significant insights into the efficacy of the curriculum requirements. It is imperative to provide comprehensive training to students to enhance the efficacy of root canal treatment. It is advisable to introduce new techniques and instruments into the curriculum to augment the quality of endodontic practice. Through theoretical training and in vitro laboratory activities, students were given a learning experience that enabled them to become competent in endodontics.

## Data Availability

The datasets created and/or analysed for the current study are not publicly accessible because ethics approval was given on the grounds that only the researchers involved in the study would have access to the identified data, but they are available from the corresponding author upon justifiable request.

## Abbreviations

RCT: Root Canal Treatment
ESE: European Society of Endodontology
RDS 424: Preclinical Endodontics
RDS 511: Clinical Endodontics
CCC: Comprehensive Care Clinics
